# Knowledge and Perception of the Use of AI and its Implementation in the Field of Radiology: Cross-Sectional Study

**DOI:** 10.2196/50728

**Published:** 2023-10-13

**Authors:** Queralt Miró Catalina, Joaquim Femenia, Aïna Fuster-Casanovas, Francesc X Marin-Gomez, Anna Escalé-Besa, Jordi Solé-Casals, Josep Vidal-Alaball

**Affiliations:** 1 Unitat de Suport a la Recerca de la Catalunya Central Fundació Institut Universitari per a la Recerca a l'Atenció Primària de Salut Jordi Gol i Gurina Sant Fruitós de Bages Spain; 2 Health Promotion in Rural Areas Research Group Gerència Territorial de la Catalunya Central Institut Català de la Salut Sant Fruitós de Bages Spain; 3 Faculty of Medicine University of Vic-Central University of Catalonia Vic Spain; 4 Data and Signal Processing group Faculty of Science, Technology and Engineering University of Vic-Central University of Catalonia Vic Spain; 5 Department of Psychiatry University of Cambridge Cambridge United Kingdom

**Keywords:** artificial intelligence, perception, knowledge, survey, digital health, radiology, public health

## Abstract

**Background:**

Artificial Intelligence (AI) has been developing for decades, but in recent years its use in the field of health care has experienced an exponential increase. Currently, there is little doubt that these tools have transformed clinical practice. Therefore, it is important to know how the population perceives its implementation to be able to propose strategies for acceptance and implementation and to improve or prevent problems arising from future applications.

**Objective:**

This study aims to describe the population’s perception and knowledge of the use of AI as a health support tool and its application to radiology through a validated questionnaire, in order to develop strategies aimed at increasing acceptance of AI use, reducing possible resistance to change and identifying possible sociodemographic factors related to perception and knowledge.

**Methods:**

A cross-sectional observational study was conducted using an anonymous and voluntarily validated questionnaire aimed at the entire population of Catalonia aged 18 years or older. The survey addresses 4 dimensions defined to describe users’ perception of the use of AI in radiology, (1) “distrust and accountability,” (2) “personal interaction,” (3) “efficiency,” and (4) “being informed,” all with questions in a Likert scale format. Results closer to 5 refer to a negative perception of the use of AI, while results closer to 1 express a positive perception. Univariate and bivariate analyses were performed to assess possible associations between the 4 dimensions and sociodemographic characteristics.

**Results:**

A total of 379 users responded to the survey, with an average age of 43.9 (SD 17.52) years and 59.8% (n=226) of them identified as female. In addition, 89.8% (n=335) of respondents indicated that they understood the concept of AI. Of the 4 dimensions analyzed, “distrust and accountability” obtained a mean score of 3.37 (SD 0.53), “personal interaction” obtained a mean score of 4.37 (SD 0.60), “efficiency” obtained a mean score of 3.06 (SD 0.73) and “being informed” obtained a mean score of 3.67 (SD 0.57). In relation to the “distrust and accountability” dimension, women, people older than 65 years, the group with university studies, and the population that indicated not understanding the AI concept had significantly more distrust in the use of AI. On the dimension of “being informed,” it was observed that the group with university studies rated access to information more positively and those who indicated not understanding the concept of AI rated it more negatively.

**Conclusions:**

The majority of the sample investigated reported being familiar with the concept of AI, with varying degrees of acceptance of its implementation in radiology. It is clear that the most conflictive dimension is “personal interaction,” whereas “efficiency” is where there is the greatest acceptance, being the dimension in which there are the best expectations for the implementation of AI in radiology.

## Introduction

Artificial Intelligence (AI) has been developing for decades, but its use in the field of health care has experienced an exponential increase in recent years. Currently, there is little doubt that these tools have come to transform clinical practice [1,2]. AI is capable of managing large amounts of information with effectiveness and efficiency beyond the reach of human capability. It is changing clinical care by improving the speed and reliability of diagnostic processes and other health-related procedures [3,4].

Although AI has been used for some time now in some areas of medical processes such as triage support [5], suggesting diagnoses from radiological scans [6] or in specialties such as ophthalmology, dermatology, pathological anatomy, or radiology [7-10], everything suggests that, in a short period of time, these tools will multiply in number and gain weight within the health care field, provided that the ethical and legislative dilemmas raised by their implementation are resolved [11-14].

As for radiology, some tools such as computer-aided diagnosis have been used in the practice of the specialty for decades now. However, with the introduction of new technologies such as deep learning*,* these tools may become much more powerful and revolutionize this field [15]. This revolution will necessarily have to be accompanied by changes in the training that radiologists receive and in their competencies, but, at the same time, it opens up a new range of opportunities for the specialty [16,17].

In recent years, most studies have focused on the perception of health care professionals regarding the implementation of AI in their practice [18], but it is also necessary to conduct studies focused on the perceptions of users and to consider user preferences to determine their limits and seek the acceptance of society [19].

A study by Ongena et al [20], focused on the field of radiology, showed that patients had little confidence in AI for diagnosis, both in terms of accuracy and confidentiality and especially in terms of personal interaction and communication. In addition, opinions on workflow improvements were ambiguous. However, they preferred AI, as it was able to look at the whole body rather than just specific parts and could report on future diseases.

Furthermore, a qualitative study focused on capturing society’s perception of the implementation of AI in health care, in general, showed that most participants agreed that the use of AI could trigger highly beneficial changes and improvements, as well as aid in making diagnoses and treatments much more effective and personalized. That being said, although the overall perception was mostly positive, the implementation of AI also raises concerns about aspects such as privacy [21].

The study conducted in Germany by Fritsch et al [22] showed that there was a good predisposition on the part of the population to introduce the use of AI in general clinical practice, but that the knowledge of this same population about AI was limited. He also highlighted some demographic groups with more reluctance, including women, elderly people, and people with a low educational level and low technological affinity. To conclude, it evidenced a strong consensus that AI should always be ultimately controlled by a health care professional and that the ultimate responsibility would be that of the health care professional [22].

A study conducted in the United States with 926 participants showed a positive expectation toward the implementation of AI in clinical practice but also revealed some areas in which the implementation of AI raised concerns. They highlighted misdiagnosis, gaps in privacy, or reduced time spent by the physician in their care. Racial and ethnic minority groups were also found to have more concerns [23].

In the modern practice of person-centered health care, it is essential to know what the perception of users is since shared decision-making and patient empowerment are 2 pillars of current health care that have replaced, or will replace in the near future, the doctor-patient paternalism existing in past decades [24].

For all these reasons, this study aims to describe the population’s perception and knowledge of the use of AI and its implementation in radiology, through a validated questionnaire, to find out which are the most accepted and problematic areas, and to identify possible sociodemographic factors related in order to develop strategies to increase acceptance and confidence in AI.

## Methods

### Study Design and Sample

A cross-sectional descriptive study was conducted through a validated, anonymous, and voluntary survey on the use of AI in the radiology setting. The survey was open to any individual in Catalonia who had received the QR code of the survey, or who had visited any Primary Care Centre (CAP) or one of the reference hospitals in the region of Central Catalonia (comprising the counties of l’Anoia, Bages, Berguedà, Osona and Moianès and with population of 525,000 habitants). The survey was open to individuals older than 18 years, between September 2022 and March 2023. Responses from individuals residing outside Catalonia were excluded through the postal code of the population of residence.

The survey could be answered in paper format or in digital format, through a QR code that led to the Microsoft 365 questionnaire. Paper sheets and posters with access to the QR were left in all the CAPs of Central Catalonia and in the participating hospital. TeleForm (version 16.5, OpenText Teleform Software) was used to create the survey in paper format and subsequently read the responses.

A minimum of 376 surveys, distributed in the study region were required, to estimate with 95% CI and a precision of 0.08 points, the values of the 4 dimensions of the questionnaire, assuming an SD of 0.75 points [20].

### Patient and Public Involvement

The patients and public were not directly involved in the design and conduct of the study due to the cross-sectional nature of the study. It was a survey of the population of Catalonia to know their perception of the implementation of AI. In this context, the population has been the main point of the research and the results reported will be important to establish strategies in the implementation. As per the plan, the study findings will be shared with the administrators of the different sites where the study was conducted to share the results with the general population.

### Ethical Considerations

The study protocol was approved by the University Institute for Primary Care Research Jordi Gol Health Care Ethics Committee (Code 20/177-PCV). The survey was completely anonymous and no respondents could be reidentified, so no informed consent was required. It was explained at the beginning of the survey that the data generated would be processed and published. No compensation was paid to those who volunteered to participate in the survey.

### Source

A validated questionnaire [20,25] was used to ascertain users’ perceptions and knowledge of AI and its use in radiology. Although the original survey contains 5 dimensions, the survey published by the authors only addresses four: “distrust and accountability” (15 questions), “personal interaction” (6 questions), “efficiency” (4 questions), and “being informed” (4 questions), all Likert-type questions (1: strongly disagree, 5: strongly agree). In addition, it contains 5 descriptive Likert-type questions (1: strongly disagree, 5: strongly agree) on the use of computers as a tool in health care.

Permission was requested from the author to use it and translate it from English into Catalan. In order to maintain the fidelity of the original survey, 2 researchers translated it independently, pooled it, and a third helped to reach a consensus in cases of discrepancies in the translation. With the Likert scoring methodology, results were obtained within a range between 1 and 5. Due to the characteristics of the survey, results closer to 5 refer to a negative perception regarding the use of AI, while results closer to 1 express that this perception is positive.

Additionally, a first sociodemographic part (sex, age, marital status, educational level, and postal code of the population of residence) and 2 questions on knowledge of AI were added. The postal code variable was categorized to obtain the rurality variable. Towns with 10,000 inhabitants or more were considered urban and towns with less than 10,000 inhabitants were considered rural [26].

### Statistical Analysis

Categorical variables have been described with absolute frequency and percentage, and continuous variables with mean and SD. In order to calculate dimensions 1-4 of the survey, we took the average of each individual’s scale scores for the corresponding questions in each dimension. Cronbach α was used to determine the validity and reliability of 4 dimensions. Typically, Cronbach α of .7 is considered indicative of good internal consistency. However, in some cases, an α of .5 or .6 may still be acceptable [27,28]. To assess the normality of the 4 dimensions, this study used skewness and kurtosis [29-31]. Typically, an absolute skewness value greater than 3 and a kurtosis value greater than 10 may indicate a potential issue with normality. West et al [32] suggested that the absolute value of skewness and kurtosis should not be greater than 2 and 7. For the bivariate analysis between the dimensions and the sociodemographic variables, the Student *t* test or ANOVA with multiple comparisons was used. The analyses were performed with R statistical software (version 4.2.1, R Foundation for Statistical Computing), and the significance level was set at 5%.

## Results

A total of 379 people responded to the survey, with 59.8% (n=226) of them being women. The mean age was 43.9 (SD 17.52) years. In addition, 56.9% (n=215) of them had a university education, 51.5% (n=177) of them lived in rural areas, and 89.8% (n=335) of them understood the concept of AI (Table 1).

**Table 1:** Descriptive analysis of the sample (N=379).

Characteristics		Values
**Gender (N=378), n (%)**		
	Female	226 (59.8)
	Male	152 (40.2)
**Age in years (N=370), mean (SD)**		**43.9 (17.5)**
	18-34, n (%)	126 (34.1)
	35-49, n (%)	102 (27.6)
	50-64, n (%)	89 (24.1)
	≥65, n (%)	53 (14.2)
**Marital status (N=379), n (%)**		
	Single	147 (38.8)
	Married	162 (42.7)
	Divorced	24 (6.33)
	Widowed	16 (4.22)
	Others	30 (7.92)
**Educational level (N=378), n (%)**		
	Does not know or no answer	4 (1.06)
	Primary	19 (5.03)
	Secondary	34 (8.9)
	Baccalaureate, vocational training	106 (28.0)
	University students	215 (56.9)
**Rurality (N=344), n (%)**		
	Rural	177 (51.5)
	Urban	167 (48.5)
**Understand what the concept of artificial intelligence means (N=373), n (%)**		
	No	38 (10.2)
	Yes	335 (89.8)

**Figure 1:**
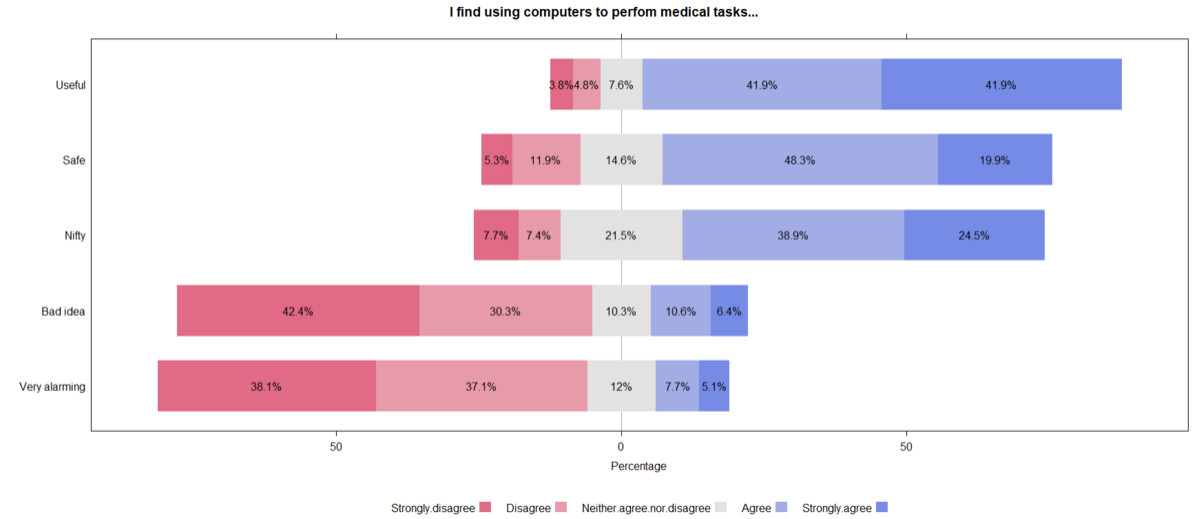
Percentage of opinions on the use of computers to perform medical tasks.

In relation to the use of computers to perform medical tasks, and considering the negative (strongly disagree and disagree) and positive (strongly agree and agree) options as a single block, Figure 1 shows that 72.7% (n=274) of the population considered that the use of computers to perform medical tasks is not a bad idea and 68.2% (n=257) thought that it is safe. In addition, 83.8% (n=316) believed that the use of computers is useful for medical tasks and 63.3% (n=239) of the sample thought it would be resourceful, while 75.2% (n=282) did not consider the use of computers in these tasks alarming.

Regarding the 4 dimensions of the survey, and each of their items, the results are expressed on a Likert scale (1: strongly disagree, 5 strongly agree), where 1 reflects positive thinking toward AI and 5 negative thinking. A preliminary analysis was carried out to estimate the internal consistency and reliability of the 4 dimensions through Cronbach α. The dimensions “distrust and accountability” and “personal interaction” obtained an estimate of 0.79 (95% CI 0.75-0.84 and 95% CI 0.75-0.82 respectively), the dimension efficacy an estimate of 0.52 (95% CI 0.42-0.60), and the dimension “being informed” an estimate of 0.42 (95% CI 0.31-0.51).

Table 2 shows the results of the dimensions and items. Following the recommendation, the analysis revealed that the absolute values of skewness and kurtosis for all dimensions were within the acceptable range of <2 and <7 respectively. The mean scores for the different dimensions were 3.37 (SD 0.53) points out of 5 for “distrust and accountability,” 4.37 (SD 0.60) points out of 5 for “personal interaction,” 3.06 (SD 0.73) points out of 5 for “efficiency,” and 3.67 (SD 0.57) points out of 5 for “being informed.”

**Table 2:** Descriptive analysis of the items and dimensions.

Dimensions			Values, mean (SD)
**Dimension 1: Distrust and accountability (N=351). 15 items; Cronbach α=.79; (95% CI 0.75-0.84)^a^**			3.37 (0.53)
	AI^b^ makes doctors lazy.	2.27 (1.14)	
	Humans have a better overview than computers on what happens in my body.	3.53 (1.16)	
	A computer can never compete against the experience of a specialized doctor.	3.44 (1.26)	
	I think the replacement of doctors by AI will happen in the far future.	2.75 (1.20)	
	I would never blindly trust a computer.	3.70 (1.23)	
	AI may prevent errors^c^.	2.13 (0.90)	
	AI can only be implemented to check human judgment.	3.21 (1.10)	
	When AI is used, my personal data may fall into the wrong hands.	3.30 (1.27)	
	I find it worrisome that a computer does not take feelings into account.	3.94 (1.20)	
	Even if computers are better at evaluating scans, I still prefer a doctor.	3.60 (1.10)	
	I think radiology is not ready to implement AI in evaluating scans.	2.85 (1.06)	
	It worries me when computers analyse scans without the interference of humans.	3.87 (1.11)	
	Through human experience, a radiologist can detect more than a computer.	3.53 (1.08)	
	It is unclear to me how computers will be used in evaluating scans	3.57 (1.08)	
	I wonder how it is possible that a computer can give me the results of the scan.	3.10 (1.22)	
**Dimension 2: Personal interaction (N=364). 6 items; Cronbach α=.79; (95% CI 0.75-0.82)^d^**			4.37 (0.60)
	Even when computers are used to evaluate scans, humans always remain responsible.	4.35 (0.89)	
	As a patient, I want to be treated as a person, not as a number.	4.51 (0.88)	
	When discussing the results of the scan, humans are indispensable.	4.37 (0.79)	
	When a computer gives the results, I would miss the explanation.	4.27 (0.94)	
	Getting the results involves personal contact.	4.10 (1.02)	
	I find it important to ask questions when getting the results.	4.58 (0.67)	
**Dimension 3: Efficiency (N=362). 4 items; Cronbach α=.52; (95% CI 0.42-0.60)^e^**			3.06 (0.73)
	Evaluating scans with AI will reduce health care waiting times^c^.	2.34 (1.01)	
	Because of the use of AI, fewer doctors and radiologists are required^c^.	3.51 (1.22)	
	As far as I am concerned, AI can replace doctors in evaluating scans^c^.	3.06 (1.19)	
	The sooner I get the results, even when this is from a computer, the more I am at ease.	3.12 (1.15)	
**Dimension 4: Being informed (N=363). 4 items; Cronbach α=.42; (95% CI 0.31-0.51)^f^**			3.67 (0.57)
	When a computer can predict that I will get a disease in the future, I want to know that no matter what.	3.82 (1.16)	
	If a computer would give the results, I would not feel emotional support.	4.25 (0.96)	
	A computer should only look at body parts that were selected by my doctor.	3.36 (1.15)	
	If it does not matter in costs, a computer should always make a full body scan instead of looking at specific body parts.	3.24 (1.35)	

^a^95% CI 3.32-3.43.

^b^AI: artificial intelligence.

^c^Items marked are recoded to measure in the same direction.

^d^95% CI 4.31-4.43.

^e^95% CI 2.98-3.13.

^f^95% CI 3.61-3.73.

Finally, the bivariate analysis between the 4 dimensions and the sociodemographic variables is presented in Table 3. In relation to the “distrust and accountability” dimension, women compared to men and people older than 65 years compared to the other age groups had significantly more distrust in the use of AI (*P*=.04 and <.01, respectively). It can also be observed that the group with university studies rated this dimension more positively than the group with baccalaureate studies and vocational training and that the population that indicated not understanding the AI concept rated it more negatively (*P*<.01 and .02 respectively).

**Table 3:** Bivariate analysis between dimensions and sociodemographic variables.

		Dimension 1		Dimension 2		Dimension 3		Dimension 4	
		Distrust and accountability, mean (SD)	*P* value	Personal interaction, mean (SD)	*P* value	Efficiency, mean (SD)	*P* value	Being informed, mean (SD)	*P* value
**Gender**			.04^a^		.17^a^		.35^a^		.95^a^
	Woman	3.29 (0.54)		4.40 (0.62)		3.03 (0.73)		3.67 (0.58)	
	Man	3.16 (0.56)		4.31 (0.57)		3.10 (0.74)		3.67 (0.55)	
**Age (years)**			<.01^b^		.91^b^		.05^b^		.05^b^
	18-34	3.38 (0.50)	a^c^	4.35 (0.58)		3.18 (0.61)		3.62 (0.58)	
	35-49	3.34 (0.52)	a	4.41 (0.57)		3.03 (0.79)		3.73 (0.54)	
	50-64	3.26 (0.57)	a	4.37 (0.56)		3.02 (0.79)		3.62 (0.62)	
	≥65	3.70 (0.45)	b^c^	4.40 (0.76)		2.85 (0.74)		3.85 (0.45)	
**Marital status**			.31^b^		.13^b^		<.01^b^		.35^b^
	Single	3.38 (0.52)		4.35 (0.61)		3.18 (0.67)	a	3.67 (0.58)	
	Married	3.36 (0.55)		4.41 (0.56)		3.04 (0.76)	a	3.71 (0.59)	
	Divorced	3.19 (0.51)		4.26 (0.42)		3.13 (0.67)	a	3.47 (0.46)	
	Widowed	3.54 (0.47)		4.03 (1.10)		2.18 (0.68)	b	3.55 (0.49)	
	Other	3.47 (0.47)		4.51 (0.49)		2.93 (0.63)	a	3.71 (0.50)	
**Level of education**			<.01^b^		.59^b^		.48^b^		.01^b^
	Does not know or no answer	3.87 (0.42)	ab^c^	4.75 (0.32)		2.62 (0.48)		4.31 (0.37)	a
	Primary education	3.55 (0.50)	ab	4.21 (0.49)		2.86 (0.66)		3.71 (0.53)	ab
	Secondary education	3.56 (0.58)	ab	4.35 (0.76)		3.04 (0.98)		3.75 (0.55)	ab
	Baccalaureate, vocational training	3.46 (0.51)	b	4.38 (0.69)		3.02 (0.74)		3.77 (0.56)	a
	University education	3.28 (0.51)	a	4.38 (0.54)		3.10 (0.70)		3.60 (0.57)	b
**Residence**			.05^a^		.27^a^		.34^a^		.24^a^
	Rural	3.43 (0.49)		4.40 (0.59)		3.01 (0.77)		3.70 (0.55)	
	Urban	3.32 (0.56)		4.33 (0.64)		3.09 (0.74)		3.63 (0.59)	
**Do you understand the concept of AI?**			.02^a^		.86^a^		.47^a^		.04^a^
	No	3.56 (0.48)		4.35 (0.56)		3.14 (0.67)		3.85 (0.53)	
	Yes	3.35 (0.53)		4.37 (0.61)		3.05 (0.74)		3.65 (0.57)	

^a^*P* value of *t* test.

^b^*P* value of ANOVA.

^c^“a”, “b”, and “ab”: Different letters indicate significant differences between groups, and groups with the same letter indicate that there are no significant differences between them. For example, for the association between dimension 1 and age, individuals aged 65 years or older had a significantly greater score as compared to individuals aged 18-34, 35-49, and 50-64 years, but there is no difference between the individuals aged 18-34, 35-49, and 50-64 years.

Regarding the dimension of “personal interaction,” there were no significant differences between the demographic characteristics analyzed, and with respect to the dimension of “efficiency” there were only differences according to marital status, with widowed users showing greater consideration of “efficiency” with respect to the rest, these being the group with the highest mean age (*P*<.01). Finally, on the dimension of “being informed,” it was observed that the group with university studies rated access to information more positively (*P*=.01) and those who indicated not understanding the concept of AI rated it more negatively (*P*=.04).

## Discussion

### Principal Findings

This study aimed to describe the level of knowledge and perception, in the population of Catalonia, of the use of AI as a health tool and its implementation in radiology. Of the 4 dimensions analyzed, “distrust and accountability” obtained a mean score of 3.37 (SD 0.53), “personal interaction” obtained a mean score of 4.37 (SD 0.60), “efficiency” obtained a mean score of 3.06 (SD 0.73), and “being informed” obtained a mean score of 3.67 (SD 0.57). In this context, the results obtained provide information on the knowledge and perception of the population and make it possible to find out which are the most problematic areas and which are the most accepted, to develop strategies to increase acceptance of the use of AI.

AI is proving to be a tool that will become fundamental in many aspects of people’s future lives and also in health care practice. In the field of diagnostic imaging, this evolution is particularly rapid and is likely to generate ethical, legal, and social conflicts over its use and acceptance [33-35]. Although patient autonomy must always be respected and any action should be individualized, knowing the population’s overall perception of the matter could help to place the patient at the center of health care. It will also be important to educate and raise awareness among both health care professionals and the general population and, in order to make these training or awareness programs more efficient, it is necessary to know in which areas there is greater distrust.

The results show that there is a high percentage of the population analyzed that has notions about the concept of AI. It must be assumed that this percentage will continue to increase since this technology is being introduced in more and more areas and is opening up to the general public, who can now make use of some of these web-based tools. The results obtained suggest an inherent resistance to the use of AI in the field of radiology, since in the 4 dimensions analyzed, a more negative assessment was obtained. Specifically, “personal interaction” was the most negatively rated dimension, while “efficiency” was the dimension in which the population analyzed was most confident.

These results are similar to those of the study conducted by Ongena et al [20] in the Netherlands and suggest that the population believes that the use of AI can improve and reduce waiting time in their medical care. However, it still raises quite a few doubts about the fact that their health care is not supervised by a human, as well as about the need for human interaction in the medical process derived from radiological studies.

Specifically, for the most negatively rated dimension, which was “personal interaction,” the results may suggest that a large part of the distrust generated by the implementation of AI in diagnostic imaging is due to depersonalization, feeling that you have not received the care you need or that the medical professional has not devoted the necessary time to your case. It is noteworthy that this fear is much more intense than the doubts that a diagnosis made by AI can cause, as evidenced by the questions “I would never blindly trust a computer,” which scores 3.70 out of 5, or “It worries me when computers analyze scans without interference of humans,” which scores a 3.87 out of 5. Moreover, this value in personal interaction remains constant across all sociodemographic groups and, therefore, reveals itself as a focal point in medical care.

Richardson et al [36] conducted 15 focus groups with adult patients who had recently visited primary care centers in order to analyze the emergence of attitudes and beliefs about health care AI. After analyzing the results, the authors proposed a conceptual framework for understanding patient attitudes and beliefs about health care AI. The attitudes and beliefs about AI used in health care are initially shaped by the patient’s past experiences. Previous illness, the use of technology in health care, the relationship between health care providers, the comfort of the patient using the technology, as well as the wider social context of the person are the main themes highlighted by patients. All of these experiences contribute to shaping the patient’s beliefs about health care and technology, which ultimately influences the development of their particular attitude toward health care AI. In this context, predicting how patients will develop an attitude toward AI in health care becomes crucial for its successful implementation.

With respect to the sociodemographic characteristics of the sample, the results obtained are noteworthy. Women and the population older than 65 years have a more negative view of the “distrust and accountability” dimension, while users with a university education have a less conflictive view of this dimension. These results are also observed in Fritsch et al’s [22] study and can be explained by the fact that the proportion of university students is likely to be lower in the 65-year-old age group. It can also be inferred that a higher level of education correlates with a higher degree of understanding and use of new technologies, which would increase confidence in them.

It has also been observed that university students are the demographic group that most positively values access to information, following the line of the study [37]. This could be linked to the fact that this more educated population group feels more capable of making and evaluating their decisions.

Furthermore, it was observed that users who indicated that they were aware of the concept of AI more positively rated access to information and the impact of AI on “distrust and accountability.” Therefore, these results may suggest that being trained or having received training may increase sensitivity to how AI can be beneficial in the health domain.

For all these reasons, the implementation of AI in the field of radiology appears to be an inexorable reality, but it must necessarily go hand in hand with acceptance by the general population taking into account cultural aspects and prior knowledge and perceptions. Studies such as the one carried out are important to take the pulse of society and design strategies to ensure that this evolution takes place under an umbrella of acceptance. Leaving users out of this process would be a mistake that could have ethical and legal consequences that we can only now begin to anticipate.

### Limitations and Strengths

As limitations of the study, we note an age bias, since the concepts used in the survey may be unfamiliar to older people. Due to this selection bias, there is more representation of younger age groups in the sample. Considering that the survey could be easily distributed and accessed via a QR code and that the subject was especially attractive among the younger population, these circumstances might have led to a higher representation of young individuals in the study sample, also resulting in a higher representation of the individuals with university level education. There could also be a selection bias due to the fact that the study population was people who had attended a health center during the study period. In addition, the survey was only translated into Catalan, the predominant language of the region studied, but with 2 official languages, Catalan and Spanish. This might have limited participation from individuals who primarily speak Spanish. Finally, there could be duplicate responses by the same user, given the web-based survey formats. While we made efforts to minimize this by using unique identifiers and tracking IP addresses, we cannot entirely rule out the chance of duplicate or fraudulent responses. This is a limitation inherent to web-based surveys. As strengths, we found a good rural or urban representation and a high pervasiveness of the AI concept that gives solidity to the results obtained.

### Conclusions

The results of the study show that the majority of the population reported being familiar with the concept of AI, with varying degrees of acceptance of its implementation in radiology. It is clear that the dimension where the population has shown the most disagreement has been “personal interaction,” while in the field of “efficiency” is where there is greater acceptance, being the dimension in which there are better expectations regarding the implementation of AI in radiology. These findings underscore the importance of considering cultural aspects, public perceptions, and knowledge when implementing AI in health care, with a focus on addressing concerns related to depersonalization and ensuring a balance between technological advancement and human interaction. This study may be helpful in creating strategies, depending on the profile of the population, to increase acceptance, reduce resistance to change, and prepare the population for a future where AI will be more and more present in health care.

## Abbreviations

AI: artificial intelligence
CAP: Primary Care Centre

## Data Availability

The data sets generated and analyzed during this study are not publicly available because the paper was based on confidential data but are available from the corresponding author upon reasonable request.
